# Influences of Climate on Phyllosphere Endophytic Bacterial Communities of Wild Poplar

**DOI:** 10.3389/fpls.2020.00203

**Published:** 2020-02-28

**Authors:** Andrea Firrincieli, Mahsa Khorasani, A. Carolin Frank, Sharon Lafferty Doty

**Affiliations:** ^1^School of Environmental and Forest Sciences, College of the Environment, University of Washington, Seattle, WA, United States; ^2^Life & Environmental Sciences School of Natural Sciences, University of California, Merced, Merced, CA, United States; ^3^Sierra Nevada Research Institute, School of Natural Sciences, University of California, Merced, Merced, CA, United States

**Keywords:** Populus microbiome, endophytes, phyllosphere, plant bacterial microbiome, xeric environment

## Abstract

Plant-associated microbial communities play a central role in the plant response to biotic and abiotic stimuli, improving plant fitness under challenging growing conditions. Many studies have focused on the characterization of changes in abundance and composition of root-associated microbial communities as a consequence of the plant response to abiotic factors such as altered soil nutrients and drought. However, changes in composition in response to abiotic factors are still poorly understood concerning the endophytic community associated to the phyllosphere, the above-ground plant tissues. In the present study, we applied high-throughput 16S rDNA gene sequencing of the phyllosphere endophytic bacterial communities colonizing wild *Populus trichocarpa* (black cottonwood) plants growing in native, nutrient-limited environments characterized by hot-dry (xeric) riparian zones (Yakima River, WA), riparian zones with mid hot-dry (Tieton and Teanaway Rivers, WA) and moist (mesic) climates (Snoqualmie, Skykomish and Skagit Rivers, WA). From sequencing data, 587 Amplicon Sequence Variants (ASV) were identified. Surprisingly, our data show that a core microbiome could be found in phyllosphere-associated endophytic communities in trees growing on opposite sides of the Cascades Mountain Range. Considering only taxa appearing in at least 90% of all samples within each climatic zone, the core microbiome was dominated only by two ASVs affiliated *Pseudomonadaceae* and two ASVs of the *Enterobacteriaceae* family. Alpha-diversity measures indicated that plants colonizing hot-dry environments showed a lower diversity than those from mid hot-dry and moist climates. Beta-diversity measures showed that bacterial composition was significantly different across sampling sites. Accordingly, we found that specific ASV affiliated to *Pseudomonadaceae* and *Enterobacteriaceae* were significantly more abundant in the phyllosphere endophytic community colonizing plants adapted to the xeric environment. In summary, this study highlights that sampling site is the major driver of variation and that only a few ASV showed a distribution that significantly correlated to climate variables.

## Introduction

The bacterial plant microbiome is important for plant growth and health, increasing nutrient acquisition (Knoth et al., 2014; Pankievicz et al., 2015; Alori et al., 2017; Chhabra and Dowling, 2017), improving abiotic stress tolerances (Cura et al., 2017; Lata et al., 2018; Zhang et al., 2019), protecting against pathogens (Bulgarelli et al., 2013; Pandey et al., 2019), modulating plant hormones (Santoyo et al., 2016; Ali et al., 2017), and detoxifying environmental pollutants (Afzal et al., 2014; Hussain et al., 2018). There is a strong and steadily increasing interest in microbial endophytes of plants (Rho et al., 2017) and how they could be harnessed to improve sustainability in agriculture, forestry and bioenergy production (Busby et al., 2017; Doty, 2017). Endophytes from plants in high stress environments have strong impacts on plant stress tolerance (Timmusk et al., 1999; Rodriguez et al., 2004; Aghai et al., 2019). While shifts in microbiome composition has been observed to be cultivar/species-specific and possibly linked to plant physiology (Perez-Jaramillo et al., 2018; Liu et al., 2019), plants can select their microbiome (Jones et al., 2019), and under abiotic stress conditions such as in drought, they have a different microbiome (Xu et al., 2018; Cheng et al., 2019). A comprehensive plant microbiome analysis of perennial species in natural environments under challenging conditions may reveal the key microbial contributors to plant stress tolerance.

Poplar (*Populus*) and willow (*Salix*) trees of the Salicaceae have a wide global distribution, both in native riparian forests across the Northern Hemisphere and in planted forests, accounting for more than 95 million hectares globally (fao.org). Native poplar trees have a diverse microbiota, many with the ability to fix dinitrogen gas, solubilize phosphate, and promote plant growth and health especially under abiotic stresses such as drought and nutrient limitation (Doty et al., 2005, 2009; Xin et al., 2009; Khan et al., 2012, 2015, 2016; Kandel et al., 2015, 2017; Doty, 2016; Aghai et al., 2019). Beneficial microbiota have been isolated from hybrid poplar trees grown in contaminated sites, in field sites, or in tissue culture (Moore et al., 2006; Ulrich et al., 2008; Barac et al., 2009; Scherling et al., 2009; Taghavi et al., 2009). Several bacterial microbiome studies were conducted from hybrid poplar or planted poplar (Bonito et al., 2014; Hacquard and Schadt, 2015; Beckers et al., 2017) while few studies have been done on native poplar in natural environments (Gottel et al., 2011; Shakya et al., 2013). Consequently, the abiotic factors that drive the variation of the phyllosphere endophytic community are still poorly understood. To our knowledge, no comparisons of the phyllospheric, bacterial microbiome of the same poplar species across environmental gradients have been reported yet.

We chose to sample black cottonwood (*Populus trichocarpa*) trees from its natural habitat range from the western and eastern slopes of the Cascade Mountains in Washington State since the mountain range creates a natural barrier separating a maritime climate on the west from a continental climate on the east (Mathews, 2016). While black cottonwood (poplar) is present across this range, there are distinct phenotypic variations and productivity across this gradient from the cooler, moister (mesic) west side to the warmer, drier (xeric) east side of the Cascades (Dunlap and Stettler, 1996, 1998, 2001). Poplar trees from the maritime, mesic climate tend to grow larger, set leaf bud later and flush earlier, have larger leaf areas and higher rust resistance compared to poplar trees from the continental, xeric climate (Dunlap and Stettler, 2001). Poplar in the xeric Yakima river valley tend to be slower growing, have greater drought resistance, and have smaller and thinner leaves (Dunlap and Stettler, 2001). The riparian zones in these river valleys are characterized by nutrient-limitation, most dominated by primary substrate, cobble and sand, deposited from the natural flooding cycles of high alpine snow melt. To determine if a core bacterial microbiome is associated with a specific ecological niche, the phyllosphere endophytic community associated to poplar branches from six river valleys across the Cascade Range was characterized.

## Materials and Methods

### Sampling and Climate Data Collection

In September 04–23, 2014, branch samples were collected from black cottonwood (*Populus trichocarpa* Torr. and Gray) trees inhabiting Yakima, Tieton, Teanaway, Snoqualmie, Skykomish and Skagit river valleys (Supplementary Figure S1A). The geographical coordinates and of each plant are reported in Supplementary Table S1. The Skagit, Snoqualmie, and Skykomish Rivers are located in the west side of the Cascade mountain range, at elevations of 45 to 200 meters. These three mesic sampling sites had cobble and sand substrates with no soil, with coniferous forest outside of the flood plain. The Tieton and Teanaway River sampling sites were on the east side of the Cascade mountain range at elevations of 567–740 m and 680 m, respectively. The Yakima River sampling sites, at an average elevation of 404 m, were distinctly xeric, with typical shrub-steppe as the accompanying vegetation. Thirty years climate, 30 days weather data and weather data at the sampling date were collected from the PRISM database^1^. Climate data are reported in Supplementary Table S1. Examples of the different environments of each river valley are shown in Supplementary Figures S1B,C.

Twig samples were placed in sterile 50 mL conical tubes and transported to the laboratory on ice and stored in a −80°C freezer. A total of 34 plants i.e., biological replicates were sampled; 6 biological replicates were collected from Skagit, 6 biological replicates were collected from the Skykomish, 3 biological replicates from the Snoqualmie, 4 biological replicates from the Teanaway, 5 biological replicates from the Tieton, and 10 biological replicates from the Yakima. For each plant, multiples twigs were collected from fully developed branches far from trunk at the 1*–*2 meter level from the ground.

### DNA Extraction, Amplification and Sequencing

Leaves from branch cuttings were surface sterilized as described (Doty et al., 2016). Surface sterilized samples were ground with mortar and pestle in liquid nitrogen to a fine powder. Total DNA was extracted from 100 milligrams of homogenized using the MasterPure Plant Leaf DNA Purification Kit (Epicentre). The quantity and purity of DNA extracts were determined with a Nanodrop ND-1000 Spectrophotometer (Thermo Fisher Scientific Inc.). Thirty ng of total DNA were used as template for PCR amplification (Supplementary Table S2) of the V4 region of the 16S rDNA gene using the primer set 515F (5′-GTGCCAGCMGCCGCGGTAA-3′) and 806rB (5′-GGACTACNVGGGTWTCTAAT-3′) (Caporaso et al., 2012), along with 100 μM of sequence-specific peptide nucleic acid (PNA) clamps for to reduce host-derived contaminations from chloroplast and mitochondria (Lundberg et al., 2012). The Exo-SAP-IT kit (Affimatrix) was used to clean the PCR products, and amplicons were tagged with Illumina sequencing primers following the standard Illumina protocol for amplicon library preparation. The libraries were then sequenced on the Illumina MiSeq sequencer using the v2 2 × 300 bp read kit by the Joint Genome Institute.

### Sequencing Data Processing and Identification of Amplicon Sequence Variants (AVSs)

Adapter and primers were removed with Cutadapt v2.4 (Martin, 2011). To identify AVSs, paired-end reads were processed using dada2 as implemented in qiime2 v2019-08 (Callahan et al., 2016; Bolyen et al., 2019). Quality trimming, denoising, merging, and chimera detection were done using the qiime2 v2019-08 plugin “qiime dada2 denoise-paired” with default setting except for “–p-trunc-len-f” and “–p-trunc-len-r” which were set at 230 and 200, respectively. The resulting ASVs were taxonomically classified using the qiime2 v2019-08 plugin “*qiime feature-classifier classify-sklearn*” with the pre-trained Naive Bayes SILVA classifier v132 trimmed to the V4 region of the 16S rDNA gene (Quast et al., 2013). Finally, the plugins “*qiime taxa filter-seqs*” and “*qiime taxa filter-table*” were used to filter out ASVs taxonomically affiliated to “chloroplast” and “mitochondria.”

### Statistical Analysis

Core microbiome analysis were performed using the R package Microbiome v1.9.19^2^. For alpha diversity measures, each sample was rarefied down to 15,000 sequences. For analyses other than alpha diversity, a normalization method for zero-inflated sequencing data (GMPR) was used (Chen et al., 2018). The function “*estimate_richness*” from the R package “phyloseq v1.22.3” (McMurdie and Holmes, 2013) was used to estimate Chao1 and Shannon alpha-diversity measures. A non-parametric Wilcoxon sign rank test was used to compare alpha-diversity indices between sites. Differences across sites were considered significant for adjusted *P*-value < 0.1 (Benjamin-Hochberg method). A Principal-coordinate analysis (PCoA) based on Bray-Curtis dissimilarities was computed using the “ordinate” function implemented in “phyloseq v1.22.3”. The function “adonis,” from the package vegan 2.1-10, was used to perform a permutational univariate analysis of variance on Bray-Curtis dissimilarities and calculate the contribution of sampling site and climate variables using with 999 permutations. The constrained correspondence analysis (CCA) implemented in the R package Vegan 2.1-10 was used to evaluate how climate data shapes the microbial community (Oksanen, 2011). Finally, a Pearson correlation was used to find ASV whose abundance significantly correlate with climate variables.

## Results

### Sampling Site Description

**FIGURE 1:**
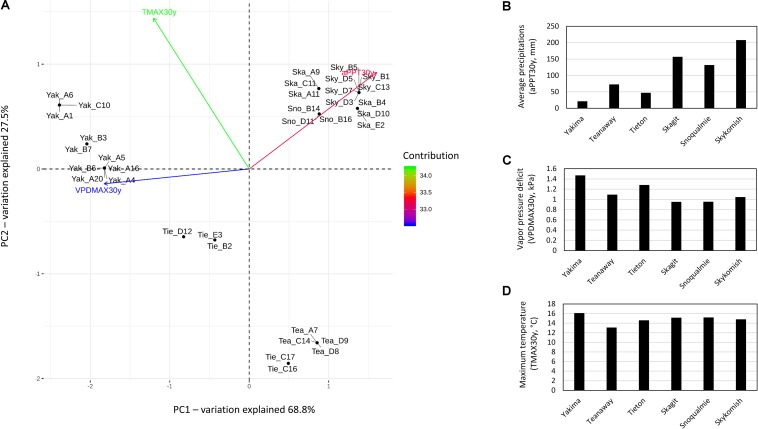
Principal component analysis and trends on 30 year climate data, max temperature, average precipitations and max vapor pressure deficit in Yakima (Yak), Tieton (Tie), Teanaway (Tea), Snoqualmie (Sno), Skagit (Ska) and Skykomish (Sky) sampling sites. **(A)** Principal component analysis if 30 year climate data; the total contribution to PC1 and PC2 of max temperature (TMAX30y), average precipitations (aPPT30y) and maximum vapor pressure deficit (VPDMAX30y) on the first and second component are encoded in color scale. **(B–D)** trends of 30 year climate data, samples are grouped by sampling sites: Yakima, Teanaway, Tieton, Skagit, Snoqualmie and Skykomish.

According to 30 years climate data i.e., maximum vapor pressure deficit (VPDMAX30y), maximum temperature (TMAX30y) and average precipitation (aPPT30Y), all variables equally contributed to the separation of sampling sites into three distinct climatic zones i.e., hot-dry (Yakima River), riparian zones with mid hot-dry (Tieton and Teanaway Rivers) and moist-cool (mesic) climates (Snoqualmie, Skykomish and Skagit Rivers) (Figure 1A). aPPT30y and VPDMAX30y reaching the highest values in moist-cool (Skykomish, Skagit, Snoqualmie) and hot-dry (Yakima) sites, respectively (Figures 1B,C) while TMAX30y reached the lowest values in mid hot-dry riparian zones (Teanaway and Tieton) (Figure 1D). Therefore, plant inhabiting hot-dry climates are subjected to drought conditions as a consequence of low precipitation and high temperature which causes high levels of vapor pressure deficit.

### Microbial Composition and Alpha Diversity of the Endophytic Community

A total of 4,181,531 paired-end reads, with an average of 122,986.2 reads per sample, were generated. After quality filtering, denoising, merging and chimera screening an average of 97,664.5 reads per sample were obtained (Supplementary Table S3). Because bacterial 16S rDNA primers also target chloroplast and mitochondrial DNA, the actual number of amplicons per sample representing the phyllosphere community ranged from 15,739 to 100,397. After the removal sequences affiliated to chloroplast and mitochondria, 587 amplicon sequence variants (ASV) were identified (Supplementary Table S4). Despite a large fraction of amplicons assigned to plastid 16S rDNA, sequencing depth was high enough to capture the majority of observed ASV (Supplementary Figure S2).

**FIGURE 2:**
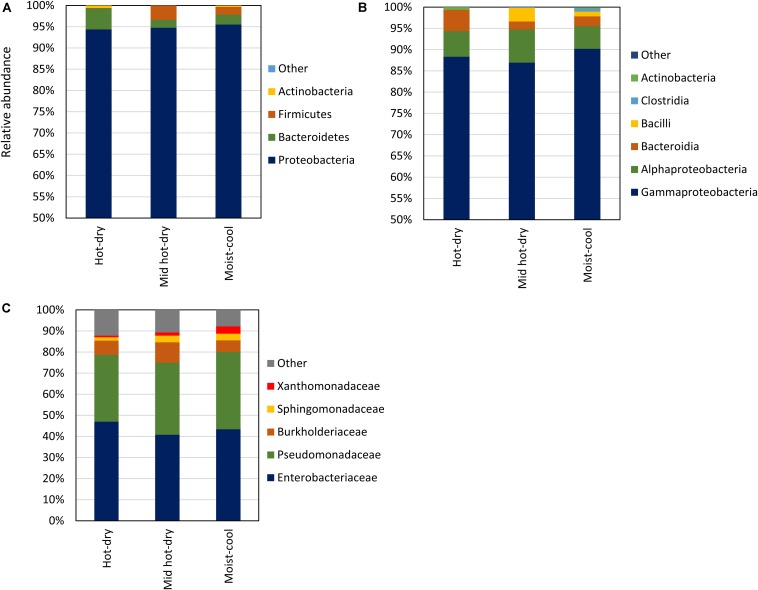
Library composition based on the taxonomic classification of the 16S rDNA sequence variants of phyllosphere endophytic bacterial communities collected in hot-dry, mid hot-dry and moist-cool climatic zones. Taxonomy is displayed at Phylum **(A)**, Class **(B)**, and Family **(C)** level. Average values of relative abundance per climatic zone: hot-dry (*n* = 10), mid hot-dry (*n* = 9) and moist-cool (*n* = 15) are plotted. Relative abundance per samples are reported in Supplementary Table S4.

Considering taxa with a relative abundance > 1.0% in at least 2 samples, the endophytic microbiota consisted of *Proteobacteria, Bacteroidetes, Firmicutes* and *Actinobacteria*. Proteobacteria was the dominant phylum, ranging from 69 to 99.9% of the total relative abundance, followed by *Bacteroidetes* (0.01–30%), *Firmicutes* (0.01–26%), and *Actinobacteria* (0.01–4%) (Figure 2A and Supplementary Table S5). Only few sequences, in total 148, were not assigned to any phylum. *Proteobacteria* were exclusively represented by *Gamma* and *Alphaproteobacteria*; *Bacteroidia* was the only class detected in *Bacteroidetes*, while Firmicutes were represented by *Bacilli* and *Clostridia* (Figure 2B). The latter were detected only in two samples. At finer taxonomic levels, only a few families occurred with relative abundance of more than 1% across all samples. Among these, *Pseudomonadaceae* (*Gammaproteobacteria*) and *Enterobacteriaceae* (*Gamma- proteobacteria*) dominated all samples, accounting all together for the 94 – 22% of the microbial community, followed by *Burkholderiaceae* (*Gammaproteobacteria*; aka *Betaproteobacteria*), *Sphingomonadaceae* (*Alphaproteobacteria*) and *Xanthomonadaceae* (*Gammaproteobacteria*) (Figure 2C). All *Pseudomonadaceae* ASV were affiliated to the *Pseudomonas* genus, while only few ASV belonging to *Enterobacteriaceae* were classified down to the genus level (Supplementary Table S4).

For each climatic zone, hot-dry, mid hot-dry and moist, a core microbiome was computed by selecting features with a relative abundance ≥1% within each sample and setting 50% occurrence as minimum threshold (Table 1). The most abundant core ASVs were affiliated to the genus *Pseudomonas* and *Enterobacteriaceae* family. At 90% threshold the core microbiome within each sampling site was dominated by ASV5 (*Pseudomonas*) and ASV17 (*Enterobacteriaceae*) (Table 1). We attempted to classify ASV17 and ASV5 down to species level by aligning the 16S sequences against all *Enterobacteriaceae* and *Pseudomonas* currently available in the Integrated Microbial Genome database. Interestingly, ASV17 generated significant alignment (100% sequence identity) with *Serratia*/*Yersinia*/*Rahnella* strains while ASV5 shared 100% identity with the 16S of *Pseudomonas viridiflava*. At lower threshold, 50–80% occurrence, other *Enterobacteriaceae* and *Pseudomonadaceae* ASVs were included part of the core members of each climatic zone. The only exceptions were represented by *Duganella*, *Xanthomonas* and *Sphingomonas* ASVs which occurred only in mid hot-dry and moist climatic zones (Table 1). Therefore, despite different climatic conditions, all samples shared ASVs mainly affiliated to *Enterobacteriaceae* and *Pseudomonas*.

**TABLE 1:** Core microbiome ASVs detected in the phyllosphere endophytic community of poplar plants inhabiting hot-dry, mid hot-dry and moist-cool climatic zones.

Climatic zone	Sequence variant ID	Occurrence	Taxonomy^1^	Number of samples	Relative abundance (%)
Mid hot-dry (*n* = 9)	ASV5	1.00	*Pseudomonas*	9	2.11–12.65
	ASV428	1.00	*Pseudomonas*	9	1.33–12.26
	ASV17	1.00	*Enterobacteriaceae*	9	4.35–38.84
	ASV1	0.89	*Enterobacteriaceae*	8	0.56–51.09
	ASV11	0.67	*Pseudomonas*	6	0.09–29.55
	ASV2	0.67	*Pseudomonas*	6	0.1–4.71
	ASV8	0.67	*Duganella*	6	0.0–12.35
	ASV147	0.67	*Sphingobium*	6	0.0–4.42
	ASV233	0.55	*Pseudomonas*	5	0.0–19.07
Hot-dry (*n* = 10)	ASV5	0.90	*Pseudomonas*	9	0.72–45.67
	ASV1	0.80	*Enterobacteriaceae*	8	0.0–49.93
	ASV2	0.60	*Pseudomonas*	6	0.0–28.53
	ASV354	0.50	*Enterobacteriaceae*	5	0.0–22.56
Moist-cool (*n* = 15)	ASV17	1.00	*Enterobacteriaceae*	15	9.84–44.98
	ASV5	0.93	*Pseudomonas*	14	0.44–53.36
	ASV439	0.80	*Pectobacterium*	12	0.0–22.48
	ASV1	0.73	*Enterobacteriaceae*	11	0.0–15.10
	ASV41	0.73	*Xanthomonas*	11	0.07–12.72
	ASV233	0.67	*Pseudomonas*	10	0.0–16.08
	ASV8	0.53	*Duganella*	8	0.0–6.33

*^1^Full taxonomy lineage is reported in Supplementary Table S4.*

### Alpha Diversity Analysis

**FIGURE 3:**
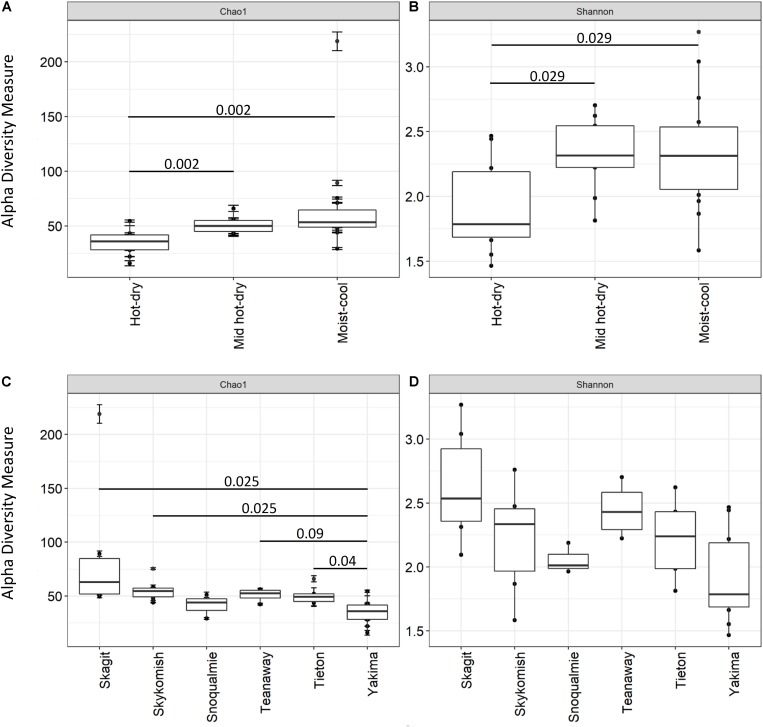
Comparison of Alpha diversity indices Chao 1 and Shannon across hot-dry, mid hot-dry and moist-cool climatic zones **(A,B)** and between sampling sites **(C,D)**, i.e., Yakima, Skagit, Tieton, Teanaway, Snoqualmie, Skykomish. Only significant Benjamini–Hochberg adjusted *P*-values are shown. For completeness all adjusted *P*-value from pairwise comparison were added to Supplementary Table S6.

Shannon and Chao 1 indices were used to measure the Alpha diversity of the endophytic community (Figure 3). All diversity metrics tended to be significantly higher (adjusted *P*-value < 0.1) for the phyllosphere community of plants colonizing moist-cool and mid hot-dry environments (Figures 3A,B and Supplementary Table S6). Similarly, although no significant differences were observed in microbiome diversity, when Shannon and Chao 1 indices were compared across sampling sites, phyllosphere community associated to plants inhabiting Tieton, Teanaway, Skagit, Skykomish and Snoqualmie river systems tended to show higher species richness and diversity compared to Yakima samples (Figures 3C,D and Supplementary Table S6).

### Community Structure as a Function of Environmental Characteristics

**FIGURE 4:**
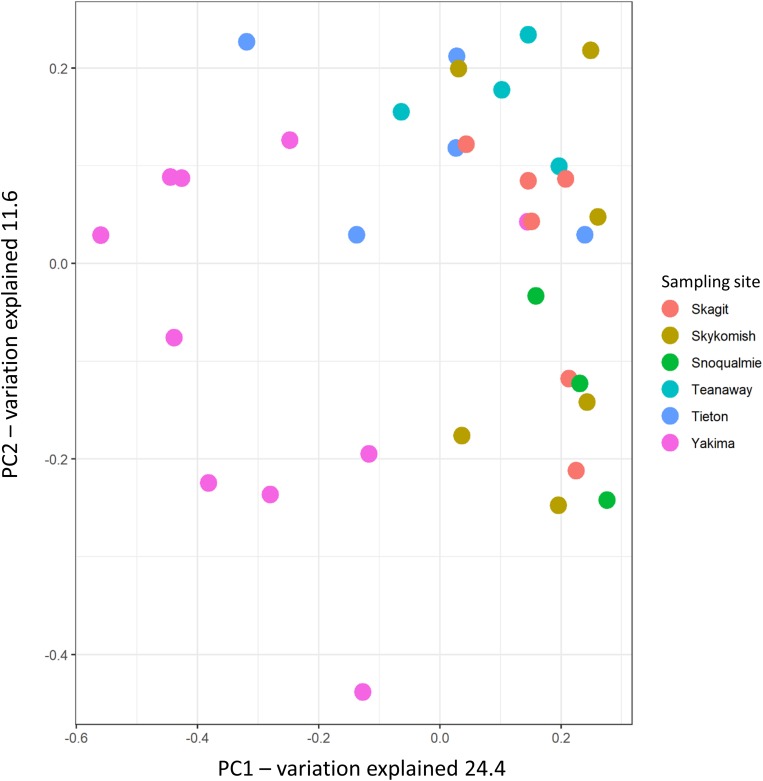
Principal coordinate analysis of phyllosphere endophytic microbial communities using Bray-Curtis distances. Circles represent samples.

The variation partitioning on Bray–Curtis dissimilarity was calculated to assess how sampling site, climate and weather variables differentiated the phyllosphere endophytic community across sampling sites. As expected, from Bray–Curtis dissimilarity distances calculated on GMPR-normalized microbial abundance data (Supplementary Table S4), site was the strongest driver of bacterial community variation, explaining 29.01% of the variance (*P* = 0.001) (Figure 4 and Table 2). Weather data collected at the sampling date had a low impact on community structure variation; precipitation and max temperatures explained, respectively the 6.1% (*P* = 0.025) and 6.2% (*P* = 0.02) of variation while the effect of vapor pressure deficit was not significant (*P* = 0.052). On the other hand, the effects of 30 days weather and 30 year climate variables on Bray–Curtis was greater compared to weather data measured at the sampling date (Table 2). Notably, among 30 days weather data vapor pressure deficit and max temperature showed comparable effects on variation in phyllosphere endophytic community, respectively 16.6% (*P* = 0.001) and 16.5% (*P* = 0.001), while among 30 years climate data vapor pressure deficit was the strongest driver of variation, explaining the 17% (*P* = 0.001) of the variance (Table 2).

**TABLE 2:** Bacterial community structure variation explained by weather and climate data, and sampling site (PERMANOVA on Bray-Curtis dissimilarities).

	Variables^1^	Df	*R* squared	*P*-value
Sampling date weather data	precipitation	1	0.06197	0.025
	max temperature	1	0.06208	0.02
	vapor pressure deficit	1	0.05223	0.052
30 days weather data	precipitation	1	0.08801	0.003
	max temperature	1	0.16602	0.001
	vapor pressure deficit	1	0.16523	0.001
30 years climate data	precipitation	1	0.1321	0.001
	max temperature	1	0.0986	0.001
	vapor pressure deficit	1	0.17099	0.001
Sampling site	–	5	0.29012	0.001

*^1^Each variable was tested individually.*

**FIGURE 5:**
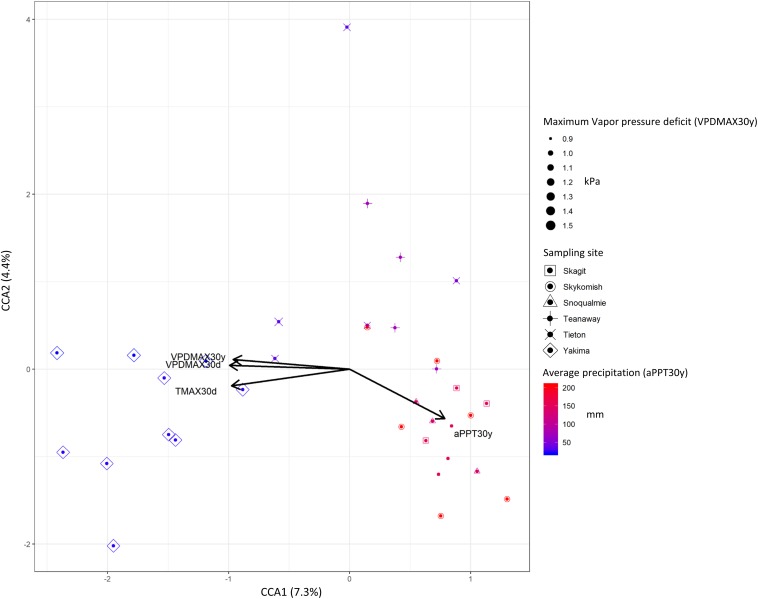
Canonical constrained analysis (CCA) biplot relating community composition to climate and weather variables. The percent of variation explained by each axis is indicated in parentheses. Symbols size and color gradient represents 30 years climate data maximum vapor (VPDMAX30y) pressure deficit and average precipitations (aPPT30y).

To better capture the variation explained by those variables that mostly affected beta-diversity (*P* < 0.0; *R*^2^ > 0.1), we performed a constrained correspondence analysis (CCA) on normalized abundance data. Accordingly, 7.3% was the total variance explained by those variables that had major effects on beta-diversity, contributing to the separation of phyllosphere community of plants growing in hot dry zones from those inhabiting mid hot-dry and moist climates (Figure 5). Therefore, we sought to determine which ASVs significantly correlated with these variables. As expected, due to the limited effect that climate/weather variables had on the distribution of ASVs across sites, very few of them moderately correlate with temperature, vapor pressure deficit and precipitation. Interestingly, a climate/weather-dependent distribution was observed for core ASVs. Specifically, ASV17 (*Enterobacteriaceae*), which is part of the core microbiome in mid hot-dry and moist-cool samples negatively correlate with vapor pressure deficit and temperature, while ASV1 (*Enterobacteriaceae*) and ASV2 (*Pseudomonas*), which are also part of the “core” of mid hot-dry and hot-dry samples, positively correlates with temperature and vapor pressure deficit (Table 3).

**TABLE 3:** Correlation of ASV abundance data with the 30 year climate and 30 days weather data.

Amplicon sequence variant ID	Taxonomy	30 days		30 years	
		Maximum temperature	Maximum vapor pressure deficit	Average precipitation	Maximum vapor pressure deficit
ASV41	*Xanthomonas*	−0.48**	−0.57***	0.56***	−0.61***
ASV17	*Enterobacteriaceae*	−0.49**	−0.49**	ns	−0.54***
ASV1	*Enterobacteriaceae*	0.44**	0.46**	−0.52**	0.52**
ASV2	*Pseudomonas*	0.63***	0.46**	ns	0.48**
ASV417	*Allorhizobium-Neorhizobium-Pararhizobium-Rhizobium*	ns	Ns	0.51***	ns
ASV19	*Allorhizobium-Neorhizobium-Pararhizobium-Rhizobium*	ns	ns	0.48**	ns
ASV40	*Burkholderiaceae*	ns	0.46**	ns	ns

Numbers represent correlation coefficient (Pearson); Significance levels for each variable are given by: **P < 0.01; ***P < 0.001; ns, *P* > 0.05.

## Discussion

The plant microbiome plays a major role in the plant response to abiotic factors and mitigation of stresses through the induction of tolerance mechanisms via phytohormone production, improved water-use efficiency, nutrient uptake, and uptake/degradation of pollutants (e.g., heavy metals and organic pollutants) (Khare et al., 2018; Lata et al., 2018). In addition, the host could directly affect the composition of the plant-associated microbial community by modifying the chemical features of the surrounding environment (Gopal and Gupta, 2016). For instance, the ability of microbes to metabolize plant-derived metabolites e.g., root exudate, implies that the plant microbiome can vary greatly among hosts, as a consequence of plant metabolism/development, and in response to environmental cues/stressors (Naylor et al., 2017; Sasse et al., 2018). These mechanisms have been described in respect to the plant rhizobiome, the microbial community associated with rhizosphere, rhizoplane and root endosphere. Less is known about the drivers of microbiome variation in the aerial tree surface, which is characterized by being extremely poor in nitrogen and carbon sources, and subjected to more rapid fluctuations of physical conditions (Lindow and Brandl, 2003; Laforest-Lapointe et al., 2016; Remus-Emsermann and Schlechter, 2018). Environmental variables such as radiation, precipitation, temperature and humidity have a direct effect on stomata opening and, therefore, play a pivotal role in regulating CO_2_ uptake for photosynthesis. In this respect, several studies have confirmed that endophytes can affect host fitness under drought conditions having a direct effect on stomata conductivity (Elmi and West, 1995; Arnold and Engelbrecht, 2007; Khan et al., 2016; Rho et al., 2018). While these studies have been conducted under controlled conditions, the importance of the phyllosphere microbial communities in natural ecosystems is still poorly understood (Laforest-Lapointe et al., 2016, 2017).

Our study characterized the structural features of the phyllosphere microbial communities collected from *Populus trichocarpa* plants inhabiting Yakima, Tieton, Teanaway, Snoqualmie, Skykomish and Skagit riparian zones, describing the impact of environmental factors, i.e., temperature, vapor pressure deficit and precipitation, to their composition. The sampling sites were characterized by different historical drought regimes as a consequence of differences in temperature, vapor pressure deficit and precipitations. In particular, based on the 30 years climate variables, the sampling sites can be pooled in three clusters characterized by hot-dry (Yakima, 10 plants), mid hot-dry (Tieton, 5 plants; Teanaway, 4 plants) and moist (Snoqualmie, 3 plants; Skagit, 6 plants; Skykomish, 6 plants) climates (Figure 1A). Such differences in temperature, precipitations and vapor pressure deficit indicates that plants inhabiting hot dry environments are subjected to drought conditions (Yuan et al., 2019).

By 16S rDNA sequencing the composition, alpha and beta diversity indices of phyllosphere-associated microbiome were characterized. The number of taxonomic groups dominating all samples was relatively scarce. Only two families had a relative high abundance, *Enterobacteriaceae* and *Pseudomonadaceae* (Figure 2C). In addition, ASVs affiliated to these families were also part of the core microbiome of the phyllosphere endophytic community of each sampling site, confirming that members of the *Pseudomonadaceae* and *Enterobacteriaceae* are ubiquitous components of the plant microbiome (Table 1; Lindow and Brandl, 2003; Jun et al., 2016; Rufian et al., 2016; Cernava et al., 2019). All the ASVs affiliated to *Pseudomonadaceae* were exclusively represented by the genus *Pseudomonas* while only few *Enterobacteriaceae* ASV were taxonomically classified down to genus level (Supplementary Table S4)*;* unfortunately, the most abundant *Enterobacteriaceae* ASV remained unidentified at lower taxonomic levels. This could be explained by the lack of power of the hypervariable region 4 in the taxonomical identification of the *Enterobacteriaceae* genera, making the 16S rDNA V4 region unsuitable for the downstream characterization of the members belonging of this family (Greay et al., 2019). However, the most dominant *Enterobacteriaceae*, i.e., ASV17 was indentified as a possible member of the genera *Serratia/Yersinia/Rahnella* which includes species recognized as plant/human pathogens and plant beneficial bacteria as well. Therefore, their identification as the most abundant ASVs might not be surprising. Similarly, the most dominant *Pseudomonas* ASV, i.e., ASV5, showed a significant hit with *Pseudomonas viridiflava*, a multi host plant pathogen (Sarris et al., 2012). None of the genera mentioned above have been recognized as pathogens in poplar, suggesting that outside its primary host, a phytopathogen could be a common inhabitant of the microbial community without contributing to plant fitness or, perhaps, acting as beneficial bacteria.

Alpha diversity indices, Shannon and Chao-1, indicates that the phyllosphere of plants inhabiting hot-dry environments had the lowest diversity. Decreases in alpha diversity as consequence of drought have been observed for microbial communities associated to plants under drought conditions before (Mendes et al., 2013; Naylor et al., 2017; Ullah et al., 2019). In our study, we found that the number of observed species (Chao-1) and the overall diversity (Shannon) tended to be lower in twigs collected from plants in hot-dry environments i.e., Yakima (Figure 3). However, from PERMANOVA analysis performed on Bray-Curtis distances, the sampling site was the major driver of variation while climate and weather data had only a limited effect on beta-diversity (Table 2). The limited effect weather and climate data on microbial community composition was also confirmed via CCA analysis (Figure 4). A possible explanation is that other environmental constraints such as chemical and physical characteristics of soil could participate to phyllosphere differentiation across sites (Verbon and Liberman, 2016; Ullah et al., 2019). However, the effect of soil composition on phyllosphere community structure could be negligible (Grady et al., 2019). Alternatively, host-specific traits that are positively selected as a result of adaptation mechanisms toward specific environmental constraints could be a major driver of variation. Indeed, as reported in Laforest-Lapointe et al. (2016), functional traits characteristic of tree ecological strategy explained the differences in leaf community structure observed across sites.

Most of the information we have regarding the molecular mechanisms behind the beneficial role of phyllosphere-associated bacteria have been obtained from studies examining the interaction between the host plant and single strains. As mentioned before, the effect of drought on the leaf gas exchange involves a strict regulation of stomata opening, which directly affects the photosynthetic capacity of the plant (Urban et al., 2017). In this respect, volatile organic compounds produced by plant growth promoting bacteria enhance stomatal closure and reduce water loss under drought conditions (Cho et al., 2008). Such aspects have been also studied at the community level, using a well-defined microbial consortium composed exclusively by plant growth-promoting bacteria (Rho et al., 2017). We found that only few ASV showed a positive and significant correlation with vapor pressure deficit which trigger stomatal closure due to the high evaporative demand of the air (Carnicer et al., 2013; Yuan et al., 2019). As mentioned before, *Enterobacteriaceae* and *Pseudomonadaceae* ASVs, which were identified as common inhabitants of our phyllopshere microbial community, have been extensively studied for their capability to improve plant tolerance toward abiotic stresses (Kang et al., 2015; Asaf et al., 2016). *Enterobacteriaceae* and *Pseudomonadaceae* are capable of secreting secondary metabolites or produce enzymes that enhance drought tolerance. For instance, volatile organic compounds such as acetoin and butanediol elicits stomatal closure, helping the plant prevent water loss from transpiration (Cho et al., 2008) and improving drought tolerance (Saha and Bothast, 1999; Celińska and Grajek, 2009; Ji et al., 2011; Khalifa et al., 2016). In addition, *Pseudomonas, Klebsiella, Erwinia, Serratia* and *Pantoea* species are known to be ACC deaminase-producing bacteria and therefore able to regulate plant ethylene levels inducing tolerance to drought stress (Li et al., 2015; Saikia et al., 2018; Danish and Zafar-ul-Hye, 2019). Therefore, while climate and weather data had a limited impact on microbial community composition, community ASVs significantly correlated with environmental constraints such as vapor pressure deficit could enhance drought tolerance in plant inhabiting hot-dry environments.

## Conclusion

Overall, this study highlights that the phyllosphere microbial community is dominated by relatively few species and that bacterial diversity decreases in plants inhabiting hot-dry environments. However, climate and weather variables related to drought such as temperature vapor pressure deficit and precipitation had a low impact of microbial community differentiation across sampling sites as only few ASVs significantly correlated with these environmental variables. Therefore, the variation in microbial community composition observed across sites opens up the possibility that host-specific effects as a result of the adaptation to extreme environment could be the major drivers of variation observed between hot-dry, mid hot-dry and moist-cool climates. Finally, the question whether these taxa that significantly correlate with climate and weather variables are real plant helpers still remain, and a metagenome level analysis would be more informative to better differentiate from a functional point of view those ASVs that, within the same family, show different degrees of correlation with temperature and precipitation.

## Data Availability Statement

The datasets generated for this study can be found in NCBI BioProject ID PRJNA589182.

## Author Contributions

SD and MK: conceived and designed the experiments and performed the experiments. ACF and AF: analyzed the data. SD, ACF, and AF: contributed reagents, materials, analysis tools and wrote the manuscript.

## Conflict of Interest

The authors declare that the research was conducted in the absence of any commercial or financial relationships that could be construed as a potential conflict of interest.

## Footnotes

1 http://www.prism.oregonstate.edu/

2 https://github.com/microbiome/microbiome/

## Supplementary Material

FIGURE S1 Location of the six sites sampled during in September 2014 across the state of Washington **(A)** and representative photographs of the mesic (**B**, Skykomish River) and xeric (**C**, Yakima River) environments. Nearby vegetation in the mesic environment included the climate indicator species, western hemlock (*Tsuga heterophylla*), sitka spruce (*Picea sitchensis*) and western redcedar (*Thuja plicata*). Accompanying vegetation in the xeric environment included included sagebrush (*Chrysothamnus nauseosus* and *Artemisia tridentata*), bitterbrush (*Purshia tridentata*), wildroses (*Rosa* sp.), grasses (hordeae), Saskatoon serviceberry (*Amelanchier alnifolia*), and ponderosa pine (*Pinus ponderosa*).

FIGURE S2 Rarefaction curves of sequence variants for each sample. Lines are colored by sampling sites: Yakima (purple), Teanaway (dark red), Tieton (orange), Skagit (blue), Snoqualmie (dark green), Skykomish (Cyan).

TABLE S1 Sampling site, geographical coordinates, and climate data associated to each sample.

TABLE S2 PCR reaction conditions.

TABLE S3 DADA2 denoising and taxonomy filtering stats.

TABLE S4 Taxonomic affiliation of 16S rDNA sequence variants with relative and GMPR-normalized abundance data.

TABLE S5 Relative abundance of major taxonomic groups identified in phyllosphere microbial communities of Poplars inhabiting hot-dry, mid hot-dry and moist-cool climates.

TABLE S6 Benjamin-Hochberg adjusted P-value from pairwise comparisons using Wilcoxon rank sum test.

## Abbreviations

aPPT30d: 30 days average precipitation
aPPT30y: 30 years average precipitation
ASV: amplicon sequence variant
TMAX30d: 30 days maximum temperature
TMAX30y: 30 years maximum temperature
VPDMAX30d: 30-days maximum pressure deficit
VPDMAX30y: 30 years maximum pressure deficit.
