# Supplementary figures and images for: Influences of Climate on Phyllosphere Endophytic Bacterial Communities of Wild Poplar
Source: Front Plant Sci. 2020 Feb 28;11:203. doi: 10.3389/fpls.2020.00203 (PMC7058686; doi:10.3389/fpls.2020.00203)

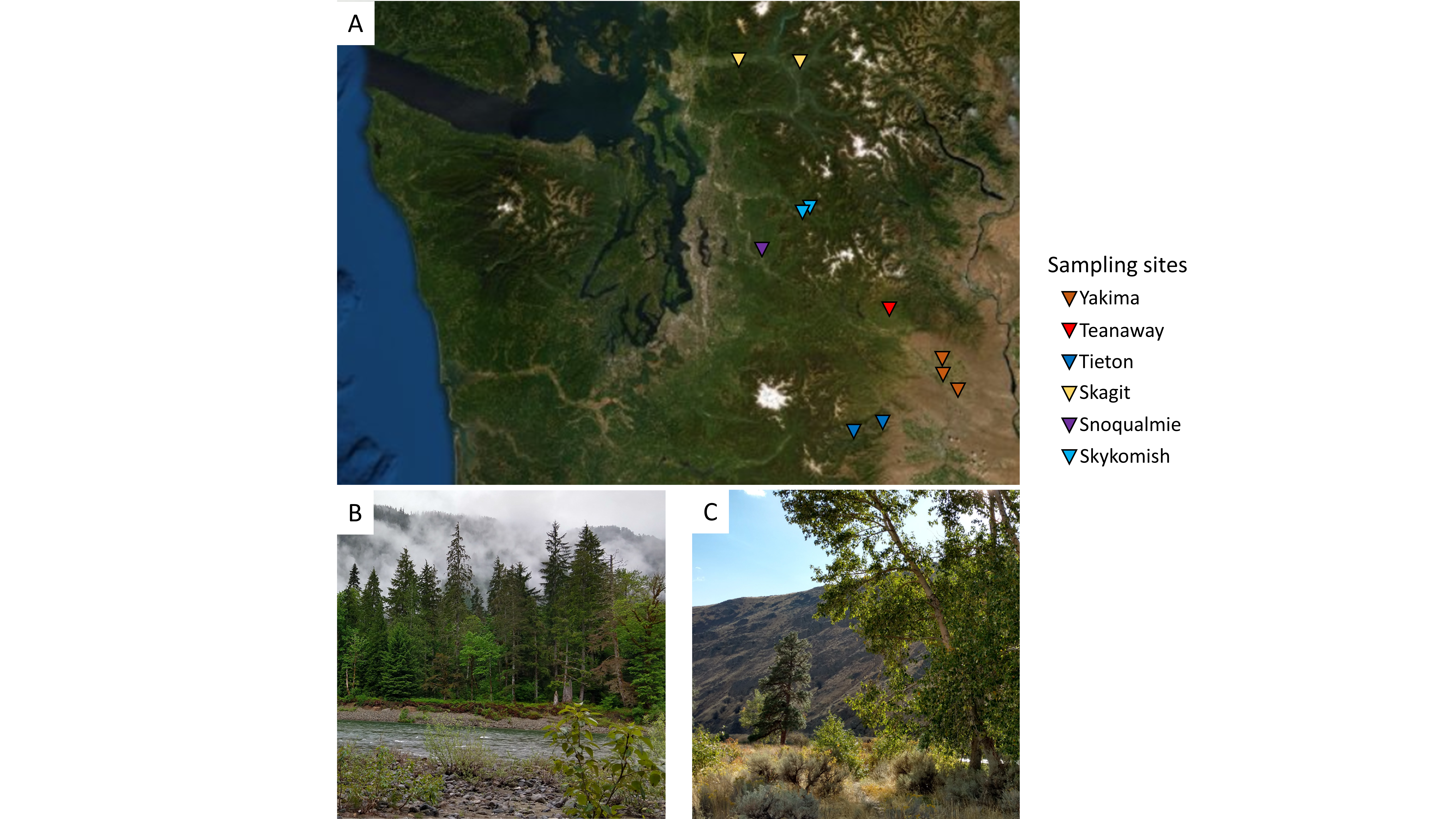

Supplement: FIGURE S1 — Location of the six sites sampled during in September 2014 across the state of Washington (A) and representative photographs of the mesic (B, Skykomish River) and xeric (C, Yakima River) environments. Nearby vegetation in the mesic environment included the climate indicator species, western hemlock (Tsuga heterophylla), sitka spruce (Picea sitchensis) and western redcedar (Thuja plicata). Accompanying vegetation in the xeric environment included included sagebrush (Chrysothamnus nauseosus and Artemisia tridentata), bitterbrush (Purshia tridentata), wildroses (Rosa sp.), grasses (hordeae), Saskatoon serviceberry (Amelanchier alnifolia), and ponderosa pine (Pinus ponderosa). [file Image_1.TIF]

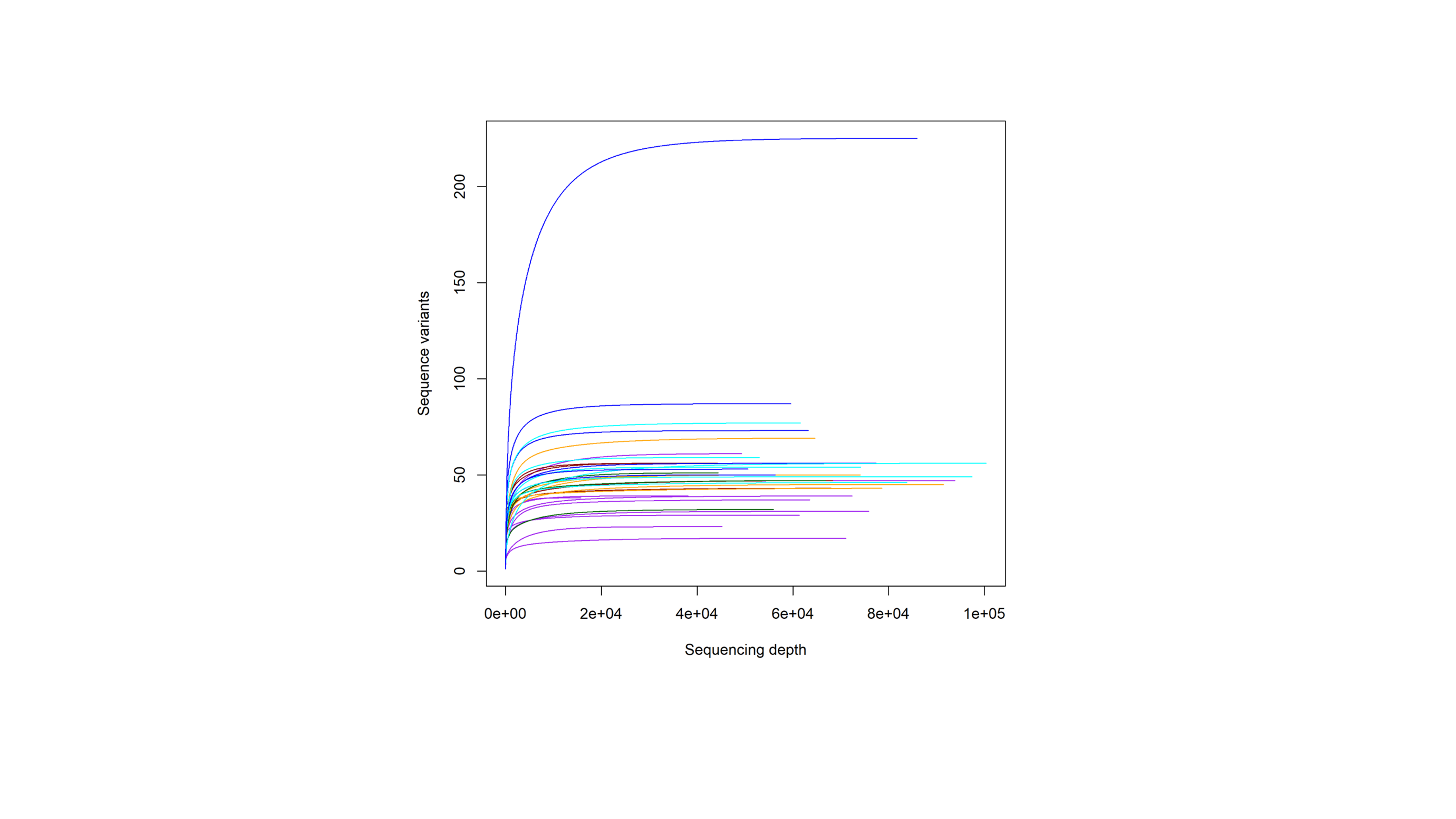

Supplement: FIGURE S2 — Rarefaction curves of sequence variants for each sample. Lines are colored by sampling sites: Yakima (purple), Teanaway (dark red), Tieton (orange), Skagit (blue), Snoqualmie (dark green), Skykomish (Cyan). [file Image_2.TIF]
